# Screening for Nephropathy in Pediatric Type 2 Diabetes: Quality Improvement to Increase Nephropathy Screening

**DOI:** 10.1097/pq9.0000000000000734

**Published:** 2024-05-27

**Authors:** Elizabeth A. Mann, Kelsi Alexander, Whitney Beaton, Elizabeth B. Roe, Amy Grant, Kristin A. Shadman

**Affiliations:** * From the Department of Pediatrics, University of Wisconsin School of Medicine and Public Health, Madison, Wisc.; † UW Health Kids, Madison, Wisc.; ‡ Cincinnati Children’s Hospital Medical Center, James M. Anderson Center for Health Systems Excellence.

## Abstract

**Background:**

Screening for early detection of microalbuminuria signaling kidney disease should begin as early as the time of diagnosis of youth-onset type 2 diabetes. This quality improvement initiative aimed to standardize urine nephropathy screening in pediatric patients with type 2 diabetes at a tertiary academic medical center and increase a baseline screening rate of 56%–75% over 6 months (September 2022–February 2023) and sustain that increase for 6 months (March through August 2023).

**Methods:**

A multi-disciplinary team used quality improvement methods and iterative Plan-Do-Study-Act cycles. Targeted interventions included previsit planning workflow, education, and a new-onset triage protocol. The team collected data at baseline and prospectively by reviewing electronic medical records. The primary outcome measure was pediatric type 2 diabetes clinic visits in diabetes clinic with urine nephropathy screening before or on the visit date.

**Results:**

A total of 121 youth were scheduled for T2D clinic visits between September 2021 and August 2023. The mean age was 14.5 years, and 60% were women, 40% were non-Hispanic Black, 28% were Hispanic/Latino, and 15% reported Spanish as their preferred language. Following the interventions of this project, urine nephropathy screening increased from 56% to 75%, and this change was sustained for 6 months.

**Conclusions:**

Interventions focused on efficient recognition of the population needing screening, coordinated internal processes around screening, a shared understanding between all stakeholders, and practical support in the healthcare system increased urine nephropathy screening with sustained improvement.

## INTRODUCTION

The incidence and prevalence of pediatric type 2 diabetes mellitus (T2D) over the last few decades has increased in the context of the childhood obesity pandemic. Based on the most recent data from the national epidemiologic SEARCH For Diabetes in Youth study, the prevalence of T2D in youth was 0.67 per 1,000 (95% confidence interval, 0.63, 0.70) as of 2017.^1,2^ The Centers for Disease Control and Prevention estimate that as many as 40,000 youths below the age of 20 years have been diagnosed with T2D.^3^ This number is increasing, as the incidence of youth-onset T2D is rising at an estimated 4.8% per year.^4,5^

Diabetic kidney disease (DKD) represents one of the most concerning microvascular complications of high glycemic levels in diabetes, accounting for increased morbidity and mortality in all populations. On a physiologic level, there are known structural changes occurring at the level of the glomerulus and tubules that can begin early in the disease process, including glomerular basement membrane thickening and mesangial expansion.^6^ Glomerular basement membrane thickening can be seen as early as 2 years following the diagnosis of diabetes and often occurs before the development of clinically significant proteinuria.^7^ These structural changes worsen over 10–20 years, and for youth with T2D, they may start to impact kidney function in late adolescence, depending on the age of onset.^8^

Children with T2D are at an increased risk of microvascular complications over their peers with T1D or adults with T2D. Compared with youth with T1D, youth-onset T2D is associated with a 4-fold increased risk of renal failure.^9^ When compared with control subjects, youth with T2D have a 23-fold risk of kidney failure and a 39-fold increased risk of dialysis.^9,10^ The Treatment Options for Type 2 Diabetes in Adolescents and Youth (TODAY) was a longitudinal clinical trial of treatment efficacy in US youth with T2D that demonstrated 8% of youth have evidence of kidney disease at T2D diagnosis and cumulative incidence was 50% within 15 years.^11^ The incidence of newly diagnosed microalbuminuria during TODAY was 10.3% over an average follow-up of 3.9 years (2.6% per year).^12^ These trends of developing T2D at younger ages raise concerns about the potential for clinically significant DKD in younger populations.^13^

Given the risk of DKD in youth-onset T2D, screening for early detection of microalbuminuria should begin as early as diagnosis. To support early diagnosis and treatment of DKD, the American Diabetes Association (ADA) recommends screening for diabetic nephropathy with urine albumin-to-creatinine ratios (UACR) at diagnosis and yearly after that.^14^ At this mid-sized academic children’s hospital, there was no standard process for ensuring nephropathy screening with UACR in youth with T2D, contributing to delayed identification of microalbuminuria in our pediatric patients with T2D. This quality improvement (QI) initiative aimed to standardize UACR nephropathy screening in pediatric patients with T2D and increase screening from a baseline of 56% to 75% over 6 months (September 2022–February 2023), and sustain that increase for at least additional 6 months (March–August 2023).

## METHODS

### Context

A multi-disciplinary team at UW Health Kids in Madison, Wisc., was formed in August 2022 to review local screening practices and baseline screening rates. With this contextual background, the improvement team sought to standardize the clinic’s approach to screening for diabetes complications in youth with T2D. This clinic provides care for approximately 100 youth with T2D annually across three clinic locations. Youth with T2D make up about 15% of the total clinic volume. Following recommendations from the ADA, youth with T2D have scheduled visits every 3 months by any of the four board-certified pediatric endocrinologists or three advanced practice providers.

### Data Collection and Reporting

The team conducted chart reviews using electronic medical records (EMR), which generated clinic visit lists (Epic, Verona, Wisc.). Data were reviewed from every scheduled visit for patients with T2D in any of the diabetes clinics. In addition to UACR order history and UACR test results, visit date and location, provider name, and basic demographic information were collected. Self-identified race and ethnicity were included to stratify our data and assess disparities between groups. We collected baseline data from September 2021–August 2022, and all variables were collected for evaluation through the completion of the project in August 2023. Chart review and data collection for this project were deemed exempt by the IRB in February 2022, as QI does not constitute research as defined under 45 CFR 46,102(d).

### Measures

The primary process measure was an up-to-date order for UACR testing in the EMR, followed by a run chart. The primary outcome measure was the percent of scheduled pediatric T2D clinic visits with urine nephropathy screening completed within the last 12 months of the visit date. The completion of screening, measured within 12 months and including visit date, was initially tracked with a run chart and later applied to a statistical process control chart. The outcome measure of completed screening was also stratified by self-identified race and ethnicity. This outcome measure was selected because it adheres to the ADA recommendations, and laboratory results are most actionable if available during the patient visit. Positive screening tests were managed according to ADA guidelines by repeating up to three times within 6 months and by referring for medication management if needed.^14^ We performed descriptive analysis on collected demographic data and one-way ANOVA analyses to compare groups. All measures were followed in real-time during the testing phase to evaluate the efficacy of changes and build understanding and support for the program.

### Testing Interventions

**Fig. 1.**
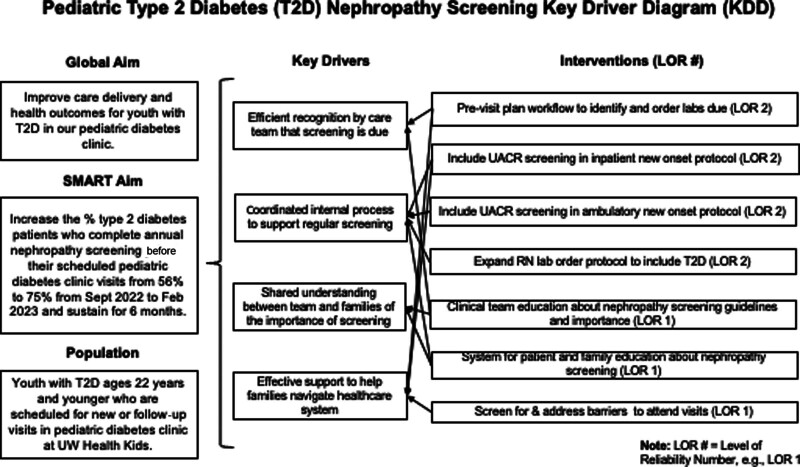
Key Driver Diagram.

The Institute for Health Improvement Model for Improvement was used to guide change theory development and testing. The team used failure mode effect analysis and Pareto charts to develop a theory for improvement and potential interventions, which they incorporated into a key driver diagram (Fig. 1). An equity lens was applied to each step in the current process to help recognize inequities that apply to each potential failure mode and ensure our proposed interventions addressed those inequities. The early Pareto analysis of missed screens suggested that a primary failure reason was the care team missing opportunities to recommend and order UACR screening. (**See figure, Supplemental Digital Content 1,** which shows the early and late Pareto charts for UACR screen. http://links.lww.com/PQ9/A558.) Thus, early interventions focused on the previsit planning process to target UACR ordering.

### PDSA Ramp 1: Previsit Planning

The first Plan-Do-Study-Act (PDSA) ramp involved creating and iterating on a previsit planning process based on published proposed frameworks.^15^ The QI project team gathered feedback of tests of change results with regular surveys and from patients and families during their clinic visits. We designed the previsit planning process to systematically identify patients due for laboratory screening, ensure screening was ordered, and facilitate completing the screening before or during the visit. All ADA-recommended laboratory testing was included in previsit planning, though this QI project specifically focused on UACR testing. Families were surveyed informally during clinic visits about the perceived value of the previsit phone call, and this informed modifications to the previsit planning template. To achieve more sustained changes, PDSA cycles tested interventions with increasing reliability numbers (LOR; Fig. 1).^16^ Applying the LOR hierarchy allowed the team to rate different tiers of intervention effort and better predict the expected outcome with each intervention. Whereas LOR 1 interventions were utilized initially, the team sought to implement changes to protocol guidance by leveraging decision aids and reminders in the process along with standardization and scheduling of key tasks (LOR 2). The finalized previsit planning process began with a care coordination assistant (CCA) reviewing an EMR-generated list of scheduled diabetes clinic visits for youth with T2D in the coming 2–3 weeks. The CCA used a template within the EMR that pulled in screening recommendations specific to that patient and any recent laboratory values, in addition to details about visit date and location identified barriers to care, and a summary of past diabetes care. If any screening was due, the CCA forwarded a request to the provider to order needed testing. Based on family preference, the CCA contacted each family before an upcoming appointment through secure messaging in the EMR or over the phone. If laboratories were due, the CCA facilitated obtaining them before the visit, if possible, or on day testing.

### PDSA Ramp 2: Education and Training

The second PDSA ramp incorporated educating care team members, patients, and their families about microvascular complications in T2D. Nursing and provider education sessions on T2D were developed and recorded for use and access virtually. Team member education included nephropathy screening guidelines from the ADA and the internal protocol for the next steps with positive screens. The improvement team developed patient and family education materials explaining the reason for urine nephropathy screening. We modified these tools with patient and family feedback during T2D clinic. PDSA cycles involved presenting education materials to five families, during routine clinic visits, along with a short three-question survey assessing readability, relevance, and perceived impact. Materials were modified based on feedback after three cycles.

### PDSA Ramp 3: New-onset Triage Protocol

Pareto analysis of missed opportunities for screening revealed that a lead cause of missed UACR screening was at initial T2D diagnosis. This may be related to differences between UACR screening recommendations for type 1 diabetes (T1D), which suggest UACR is not beneficial at diagnosis of T1D. As part of prior T2D improvement work, a new-onset T2D triage protocol had been developed. It was used by nurses and providers to guide the diagnosis and initial management of suspected T2D patients. To take advantage of the existing workflow and improve UACR screening at diagnosis, UACR was added to this protocol.

## RESULTS

A total of 121 unique patients were scheduled for T2D clinic visits between September 2021 and August 2023 (Table 1). Of all T2D patients, 60% were women, the mean age was 14.5 years (SD 2.7), 40% were non-Hispanic Black, 28% were Hispanic/Latino, and 15% reported Spanish as their preferred language. Baseline data from September 2021 through August 2022 identified 294 T2D visits scheduled in the diabetes clinic. About half of those (48%, n = 141) were completed, whereas 17% were marked no-show or late cancelation within 24 hours (n = 50). A total of 90 unique patients were scheduled during this time. During the intervention period, 98 unique individuals were scheduled for 297 visits, of which 54% were completed and 28% were marked no-show or late cancelation (n = 160 and n = 84, respectively).

**Table 1.** Demographic Data for Total Unique Patients Seen between September 2021 and August 2023

	Total (n = 121^*^)		Baseline (n = 90)		Intervention (n = 98)		
	n	Percent	n	Percent	n	Percent	*P* value
Sex							0.599
Female	73	60.3	54	60.0	58	59.2	
Male	48	39.7	36	40.0	40	40.8	
Age							0.869
6–8	3	2.5	2	2.2	1	1.0	
9–11	11	9.1	9	10.0	7	7.1	
12–14	39	32.2	31	34.4	28	28.6	
15–17	53	43.8	35	38.9	53	54.1	
18–20	15	12.4	13	14.4	9	9.2	
Race and ethnicity							0.876
Non-Hispanic Black	49	40.5	38	42.2	37	37.8	
Hispanic or Latino	34	28.1	24	26.7	30	30.6	
Non-Hispanic White	33	27.3	25	27.8	26	26.5	
Asian or Pacific Islander	2	1.7	2	2.2	1	1.0	
American Indian or Alaskan native	2	1.7	1	1.1	1	1.0	
Declines to Answer	1	0.8	0	0.0	3	3.1	
Preferred language							0.959
Spanish	18	14.9	12	13.3	17	17.3	
English	102	84.3	77	85.6	77	78.6	
Other	1	0.8	1	1.1	4	4.1	

Baseline data for n = 90 unique patients seen between September 2021 and August 2022. Intervention data for n = 98 unique patients seen between September 2022 and August 2023. *P* values were obtained using one-way ANOVA.

* Some patients in baseline analysis were also seen during intervention period so these groups are not mutually exclusive.

**Fig. 2.**
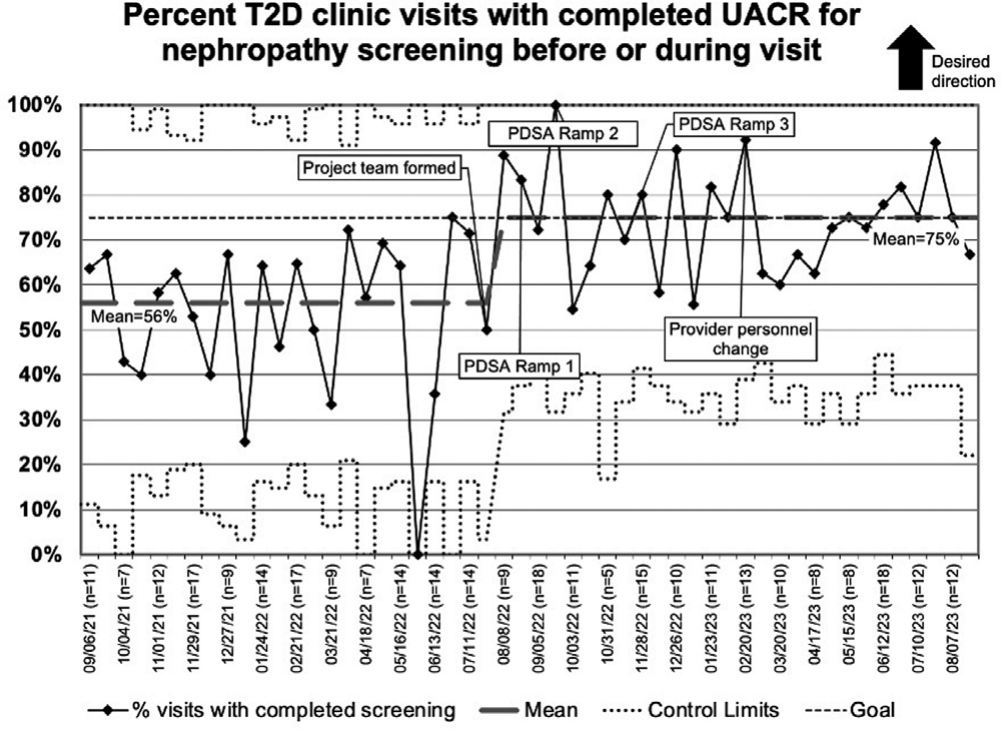
P-chart percent T2D clinic visits with completed urine albumin-to-creatinine ratio for nephropathy screening before or immediately following visit.

**Fig. 3.**
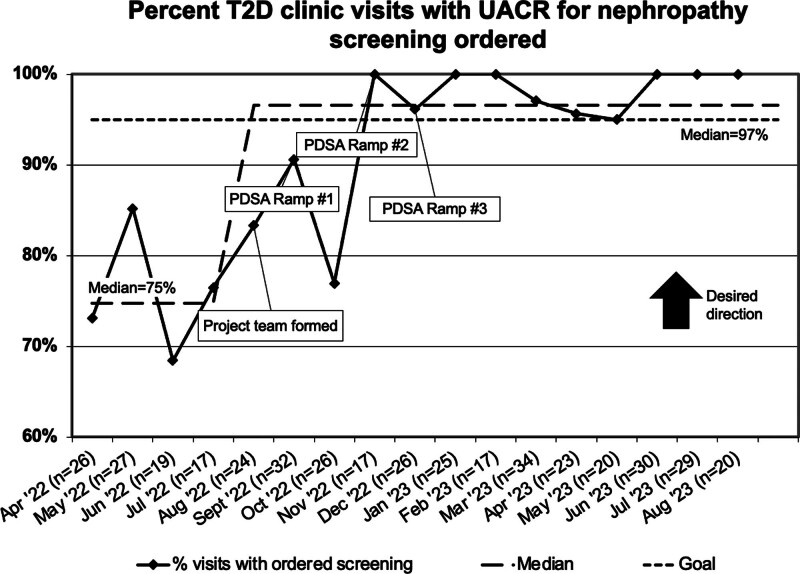
Run chart percent T2D clinic visits with urine albumin-to-creatinine ratio ordered before or during the visit.

The outcome measure of visits with UACR screening for nephropathy completed before or on the visit date is shown in Figure 2. Special cause was achieved in September 2022, shifting the centerline mean screen rate to 75%, which was sustained for the following 12 months. The process measure of the percent of visits with ordered UACR screening is shown in a run chart in Figure 3. Baseline screening rates showed order rates of 75% for the 5 months before interventions, and the project target was 95%. Order rates improved to a median of 97%, which has also been sustained. The outcome measure of screening rates was stratified by self-identified race and ethnicity throughout the project. A one-way ANOVA compared the mean screening rate of the three largest racial and ethnic groups and found no significant difference (NHB 63 ± 4%, NHW 64 ± 4%, Hispanic/Latino 72 ± 5%; *P* = 0.269).

## DISCUSSION

Microvascular complications are common in youth-onset T2D, and early recognition is critical to prevent long-term morbidity and mortality. This QI project resulted in a 30% increase in nephropathy screening rates with UACR in a pediatric T2D clinic over 6 months, with a sustained increase for over 6 months. Interventions focused on efficiently recognizing the population needing screening, coordinated internal processes to order and complete screening, a shared understanding between all stakeholders, and effective patient and family support to navigate the healthcare system.

In addition to gathering baseline data to understand the current state of screening at the beginning of this work, the improvement team used tools to outline the usual process for screening. Team members could identify the root causes of missed screening by focusing on individual failure screens. Stakeholder feedback was incorporated with Pareto analysis of missed screening which identified a “lack of recognition that screening was due” as the primary reason for a missed screen. Because of this, early interventions focused on improving processes to recognize that screening was due and that orders were needed. This audit and direct provider feedback is an interventional approach that has succeeded in other diabetes-screening QI projects. In a randomized controlled trial of internal medicine residents, increased screening of lipid and hemoglobin A1c laboratories (75.8% versus 64.1%, *P* = 0.02 and 61.5% versus 48.1%, *P* = 0.01 respectively) was seen with an intervention to audit overdue laboratories and report this to clinic providers.^17^ Similar to the success demonstrated in this study, this audit and feedback intervention successfully increased the process measure of ordering labs.

Through applying an equity lens to all possible interventions,^18^ the improvement team focused on reducing and preventing racial and ethnic disparities in screening rates. This was particularly important when designing interventions, given the disproportionately higher burden of diabetes complications in racial and ethnic minority populations in the United States.^19^ In a study of over 62,000 people with diabetes, the incidence of end-stage renal disease was higher in non-Hispanic Black, Hispanic, and Asian patients compared with non-Hispanic White patients (non-Hispanic Black 6.8 patients/1000 person-years, Hispanic 4.3 patients/1000 person-years, Asian 4.5 patients/1000 person-years, White 3.2 patients/1000 person-years).^20^ Although baseline data for this project revealed no disparity in screening rates based on race or ethnicity, interventions to prevent the disparity development were prioritized in PDSA cycles. One example involved the previsit planning process leveraging multiple methods of communication with patients and families to screen for barriers to attending clinics or the laboratory. By tracking each family’s preferred communication method (EMR-based messaging or phone) and preferred times of day for phone calls, communication could be tailored to each family. Patient and family feedback on previsit planning and education were included throughout PDSA ramps. Feedback on educational materials was incorporated throughout PDSA cycles and facilitated the development of patient-centered materials. Through this intentional approach to reducing disparity, the team accomplished overall screening improvements and did not find screening rate disparities between racial and ethnic groups.

Previsit planning is a practice that can improve value and office efficiency while being a visit satisfier for providers and patients.^21^ Previsit planning methods leveraged in this study are consistent with those most commonly used: using EMR tools to generate electronic previsit checklists and supporting patients by providing educational resources.^15^ Following the implementation of previsit planning in the study, benefits extended beyond nephropathy screening rates to include clinical efficiency. Providers experienced reduced time burden of chart review ahead of upcoming visits while maintaining perceived autonomy. This helped build support for further changes. The previsit planning process was further improved by clearly defining responsibilities and improving communication between care team members. By remaining focused on nephropathy screening, frequently communicating with the clinical team on project progress, and incorporating team member feedback, clinical team engagement remained high throughout the work.

This study has several limitations. This clinic for T2D patients has a significant no-show rate, consequently some patients are scheduled for many more visits per year than they complete, because missed appointments are often rescheduled promptly. This can skew screening rates if many patients cancel and reschedule, potentially even in the same month, as the same patient may be included more than once in the denominator of total visits. To include these patients in the screening efforts, the study team elected to include visits that were marked no-show in the screening rates. Interestingly, no differences were observed between screening rates in completed versus no-show visits in the first few PDSA cycles. Therefore, future efforts include analyses to better understand the population of patients who cannot attend clinic visits and develop interventions targeted to complete screening locally.

We set our screening target at 75% given the timeframe and baseline screen rates. While achieving this target is optimistic, a remaining challenge for the improvement team is obtaining labs for the remaining visits, with a goal of increasing screening to 95%. Despite following previsit planning steps of preordering laboratories,^22^ they still must be obtained, often outside of clinic for insurance reasons. Therefore, completion depends partly on the patients. This is a known barrier to QI interventions with diabetes screening,^23^ and will require interventions that lessen the burden on patients outside of clinic visits by improving coordination with primary care and advocating for insurance changes to support patient needs. Another limitation is that there is no control group for comparison as these QI interventions were rolled out to all patient visits. However, by following process and outcome measures over time, statistically significant changes could still be identified.

Finally, although improvement efforts included team education about recommendations for next steps in the setting of positive UACR screening tests, tracking outcomes of positive screens was out of scope for this project. Future measures will include positive screening outcomes. Though this project specifically focused on the UACR laboratory test, laboratory screening for other microvascular complications has likely improved as well, as the previsit planning incorporates all due ADA-recommended screening labs. The next steps for this improvement work are to apply a complications screening bundle that incorporates blood pressure and serum creatinine for nephropathy, as well as markers for cardiovascular disease, retinopathy, and neuropathy. By expanding screening in this way, future efforts will support the early identification of all diabetes complications.

## CONCLUDING SUMMARY

DKD is a common complication of youth-onset T2D with high morbidity and mortality.^8–10^ We achieved a 30% sustained improvement of kidney disease screening for 12 months with efficient recognition of the population due for screening, coordinated internal processes, shared stakeholder understanding, and effective patient and family support. We will apply lessons learned through this work to future efforts to increase screening further to 95%.

## Supplementary Material


