# Supplementary material for: Screening for Nephropathy in Pediatric Type 2 Diabetes: Quality Improvement to Increase Nephropathy Screening
Source: Pediatr Qual Saf. 2024 May 27;9(3):e734. doi: 10.1097/pq9.0000000000000734 (PMC11132389; doi:10.1097/pq9.0000000000000734)
Supplement: Supplementary file 1 [file pqs-9-e734-s001.pdf]

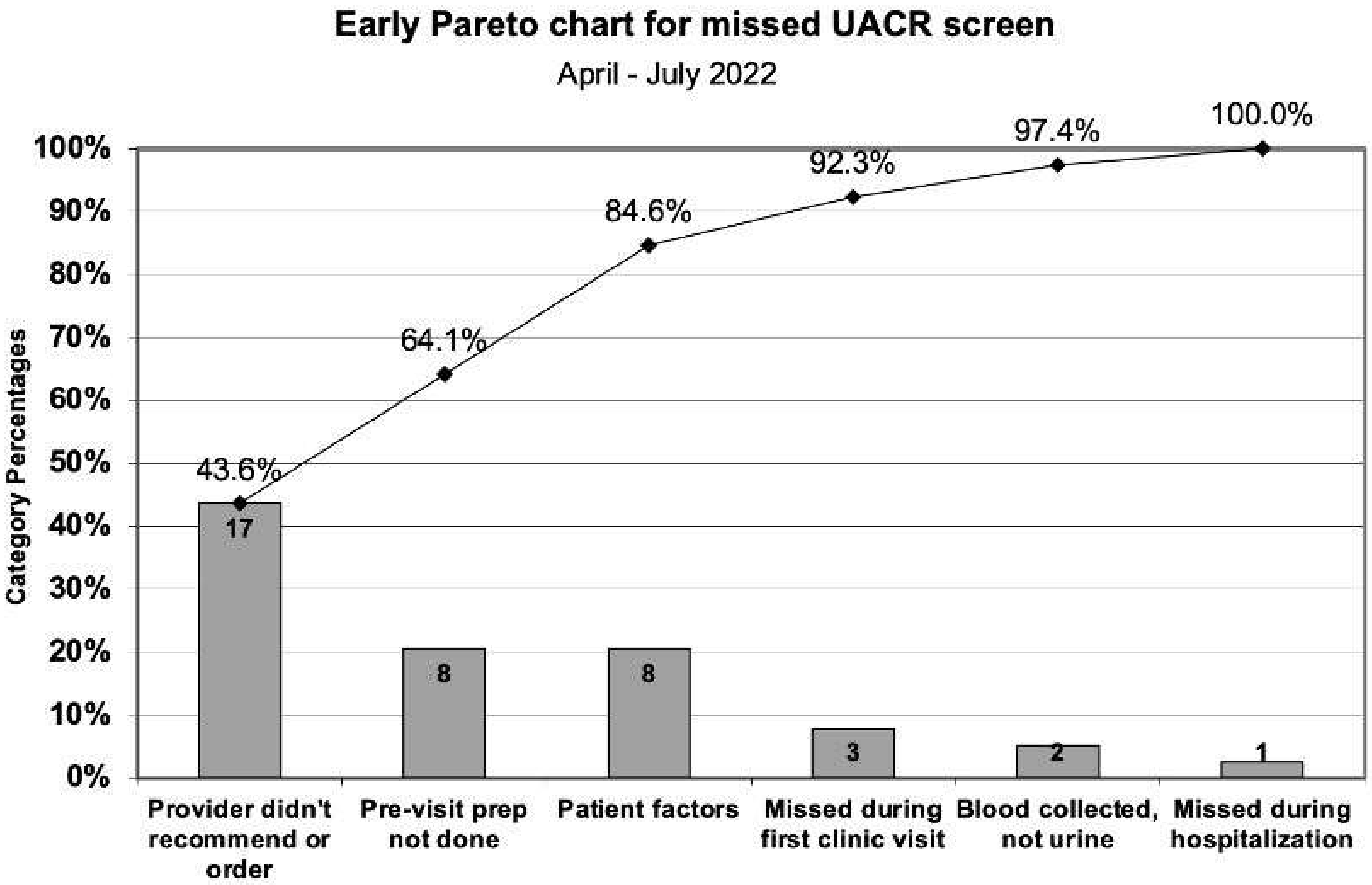

Early Pareto chart for missed UACR screen
April - July 2022
Category Percentages
100%
90%
80%
70%
60%
50%
40%
30%
20%
10%
0%
43.6%
64.1%
84.6%
92.3%
97.4%
100.0%
17
8
8
3
2
1
Provider didn't recommend or order
Pre-visit prep not done
Patient factors
Missed during first clinic visit
Blood collected, not urine
Missed during hospitalization

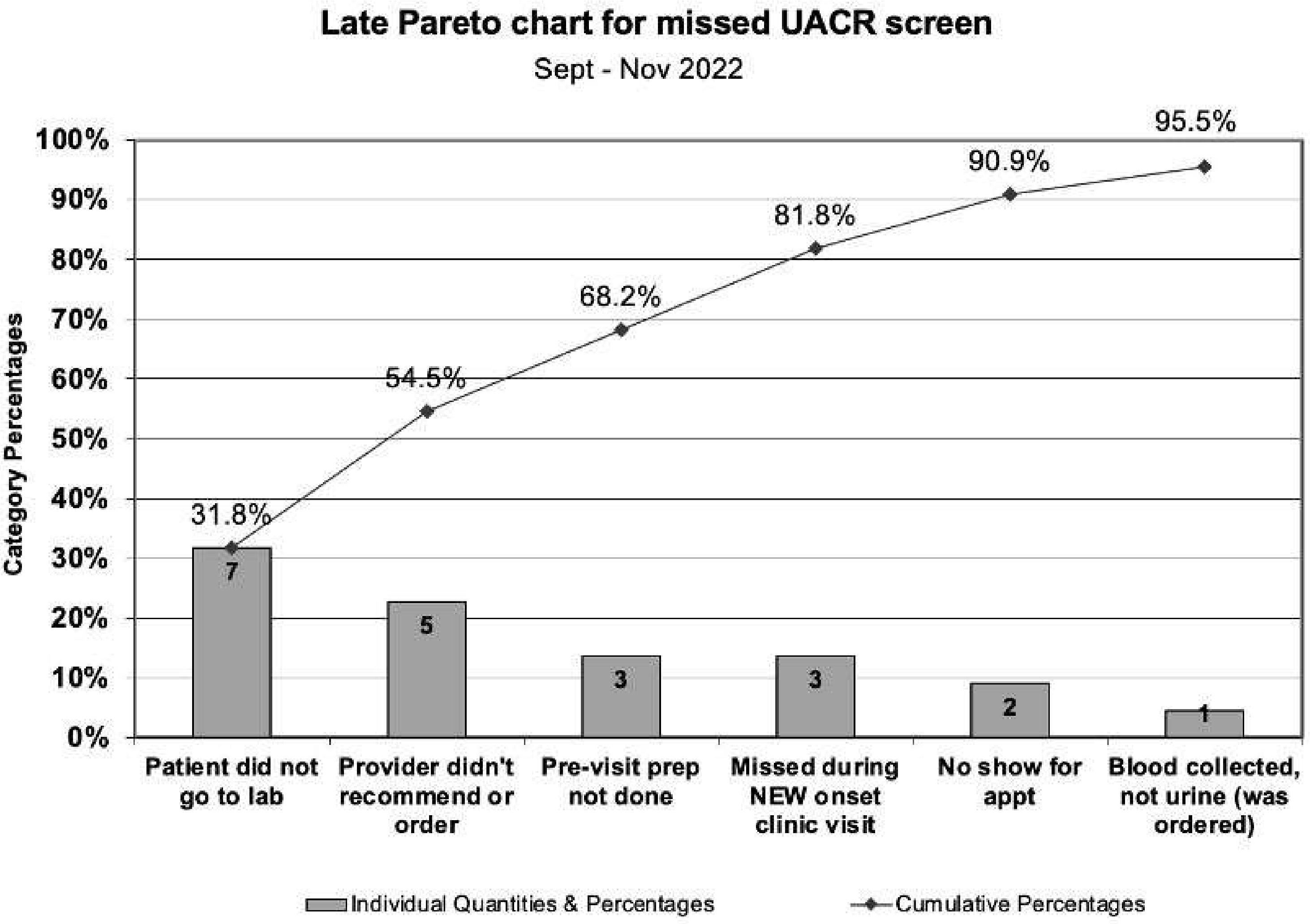

Late Pareto chart for missed UACR screen
Sept - Nov 2022
Category Percentages
100%
90%
80%
70%
60%
50%
40%
30%
20%
10%
0%
31.8%
54.5%
68.2%
81.8%
90.9%
95.5%
7
5
3
3
2
1
Patient did not go to lab
Provider didn't recommend or order
Pre-visit prep not done
Missed during NEW onset clinic visit
No show for appt
Blood collected, not urine (was ordered)
Individual Quantities & Percentages
Cumulative Percentages
